# An LGBTQ+ mentorship program enriched the experience of medical students and physician mentors

**DOI:** 10.36834/cmej.69936

**Published:** 2020-12-07

**Authors:** Rachelle A. Beanlands, Lilian J. Robinson, Shannon L. Venance

**Affiliations:** 1 Schulich School of Medicine and Dentistry, Western University, Ontario, Canada; 2 Department of Clinical Neurological Sciences, Schulich School of Medicine and Dentistry, Western University, Ontario, Canada

## Implication Statement

The Schulich School of Medicine and Dentistry LGBTQ+ mentorship program positively impacted the personal and professional experience of LGBTQ+ medical students and physician mentors. Mentorship delivered by LGBTQ+ physicians helped LGBTQ+ medical students see a positive future for themselves in the medical profession and find a trusted community of confidantes. Mentors enjoyed being positive role-models for their mentees, and felt a stronger sense of community with their colleagues. We recommend that all Canadian medical schools consider the implementation of a mentorship program designed to support the personal and professional development of their LGBTQ+-identifying students.

## Énoncé des implications de la recherche

Le programme de mentorat LGBTQ+ de la Faculté de médecine et de médecine dentaire Schulich a eu un impact positif sur l'expérience personnelle et professionnelle des étudiants en médecine et des médecins mentors LGBTQ+. Le mentorat offert par les médecins LGBTQ+ a aidé les étudiants LGBTQ+ à envisager un avenir positif dans la profession médicale et à trouver une communauté de confiance. Les mentors ont apprécié l’expérience d'être des modèles positifs pour les mentorés et ils ont éprouvé un sentiment d'appartenance plus fort entre collègues. Nous recommandons à toutes les facultés de médecine canadiennes de songer à mettre en place un programme de mentorat destiné à soutenir le développement personnel et professionnel des étudiants qui se définissent comme LGBTQ+.

## Introduction

Throughout their training, LGBTQ+ medical students experience higher rates of discrimination, isolation, and mental illness than their heterosexual, cis-gender peers.^1-3^ Mentorship is crucial to the personal and professional development of minority medical students, and there are distinct benefits when mentorship is provided to students by members of their own communities.^4^ Notably, LGBTQ+ physicians can facilitate a safe space for LGBTQ+ students to express themselves and offer guidance to those experiencing discrimination.^4,5^

Recognizing the critical importance of mentorship for minority students, we developed a novel mentorship program for LGBTQ+ medical students at the Schulich School of Medicine and Dentistry (Schulich). To understand how mentors and mentees were impacted by Schulich’s LGBTQ+ mentorship program, we distributed online surveys to participants from the 2017-2018 and/or 2018-2019 academic years and performed a qualitative analysis. Our experience implementing this group and the results of this survey inform our recommendations to other medical schools on how best to implement similar programs.

## Innovation

### Program summary

Schulich’s LGBTQ+ mentorship program is a student-led initiative for LGBTQ+ medical students. LGBTQ+ faculty and resident physicians provide mentorship to students by way of email, informal meetups, and group events. Our study was approved by the Western Research Ethics Board (WesternREM or WREM) and Lawson Health Research Institute.

### Program initiation

Prior to implementation, we received approval from Schulich’s Associate Dean of Undergraduate Medical Education (UME) and Assistant Dean Learner Equity and Wellness (LEW). The program was placed under the purview of the LEW office, which allowed us to acquire $500 of annual funding for group events.

### Program recruitment

Faculty mentors were recruited by our appointed Faculty Lead, who contacted her LGBTQ+-identified colleagues. We were provided with the names, email addresses, and specialties of interested mentors. To inform medical students of the opportunity to participate, we distributed an email and asked interested students to fill out a Google Form. Students were asked to provide their name, email, year of study, specialty of interest, and preferences for mentor gender and specialty.

### Mentorship pairings

Each mentor was paired with 2-3 medical students. We made all attempts to satisfy student requests regarding mentor gender. Mentees were introduced to their mentors via email. The group as a whole met twice yearly for social events, and individual mentors and mentees met 2-5 times each year for support, often in the mentor's home. We also circulated a list of participants and their contact information to all members of the mentorship group, with a reminder that group membership is confidential.

## Outcomes

Online surveys were distributed via email, and seven (29%) medical students and four (50%) physicians provided a response. Medical students and mentors both identified personal and professional benefits to participation.

Students felt that role-modelling by LGBTQ+ physicians provided reassurance about their capacity to achieve professional success, and they experienced a sense of belonging amongst peers and faculty (Table 1). For example, one student said, “The fact that other physicians have been out since the start of medical school and are still perfectly successful has also been reassuring.”

**Table 1:** Themes extracted from participant surveys and supporting narrative responses.

Respondent category	Extracted themes	Supporting narrative response
Medical Student	Role modelling by LGBTQ+ physicians provided reassurance about capacity to achieve professional success.	Participant 4M - “The fact that other physicians have been out since the start of medical school and are still perfectly successful has also been reassuring.” Participant 6M - “It was nice to see people of our community in positions of prominence, especially in London.” Participant 7M - “It makes it easier to envision a future for myself as a bi and trans physician, which means a lot to me.”
	Participation in a peer-led LGBTQ+ mentorship program evoked a state of shared identity and gave rise to a sense of belonging.	Participant 3M - “It has made me feel more accepted and allowed me to find classmates who I identified with.” Participant 4M - “Certainly the ability to identify others who are within the LGBTQ+ rainbow has made me more comfortable within my class.” Participant 7M - “It’s nice to know I’m not alone, and to have people who understand to talk to and vent with when faced with overt or implied prejudice.”
Physician Mentor	Identified the value of modelling personal and professional success for gender and sexual minority medical learners and gained satisfaction from doing so.	Participant 1S - “I feel that it is good for medical students, some who have not been out for long and others who are struggling with the coming out process, to see people who are ‘comfortable in their skin’ so to speak.” Participant 2S - “I feel like I have been able to provide guidance and support to both students and residents in various aspects of their academic and personal lives. I feel like I have been able to be a role model within my personal and professional life.” Participant 4S - “I am more aware that my being open about my sexual orientation can help learners and create a more inclusive environment.”
	Gained new insight into the experience of medical learners who identify as gender and/or sexual minorities.	Participant 3S - “ [I] (g)ot a better perspective of being a gay resident or medical student.”
	Fostered personal connection and professional networking opportunities with medical learners and fellow mentors.	Participant 1S - “Through the group I met a resident who is interested in coming to some of my clinics to see how I treat … and […] probably wouldn’t have known about me otherwise.” Participant 2S - “I feel a greater sense of community at Schulich.” Participant 3S - “[I] (m)et a great group of LGBTQ residents and medical students. [I] (m)et LGBTQ staff I didn’t know.”

Mentors identified the value of modelling success for gender and sexual minority students and gained new insight into the educational experience of minority learners. One mentor stated, “[...] I feel like I have been able to be a role model within my personal and professional life.” Another noted, “I am more aware that my being open about my sexual orientation can help learners and create a more inclusive environment.” Finally, physicians felt that participation fostered personal connection and led to networking with fellow mentors (Table 1).

## Next steps

Suggestions for improvement of the mentorship program included recruitment of a greater diversity of mentors, and distribution of LGBTQ+-friendly mental health resources.

Our study faced challenges in survey design, response rate, and probable response bias. Future program appraisal efforts could include qualitative research expertise and a more robust survey dissemination campaign.

Overall, participants were positively impacted by Schulich’s LGBTQ+ mentorship program. With administrative support, similar initiatives can be successful at medical schools across the country.

*An appendix describing mentorship events is available upon request*.
